# The effectiveness of face to face education using catharsis education action (CEA) method in improving the adherence of private general practitioners to national guideline on management of tuberculosis in Bandung, Indonesia

**DOI:** 10.1186/1447-056X-11-2

**Published:** 2012-03-27

**Authors:** Nita Arisanti

**Affiliations:** 1 Public Health Department, Faculty of Medicine, Universitas Padjadjaran Bandung Indonesia

## Abstract

**Background:**

In many countries, private general practitioners are the first contact in health services for people with symptoms of tuberculosis. Targeting the private sector has been recommended in previous studies to improve tuberculosis control. A brief face-to-face intervention using Catharsis Education Action (CEA) method, repeated at periodic intervals, seems to change physicians' attitudes, beliefs and practice.

The objective of the study was to determine the effectiveness of CEA method in improving the private general practitioners' (PPs) adherence to the national guideline on the management of tuberculosis patients in Bandung District, Indonesia.

**Method:**

A randomized controlled trial was done. For the intervention group, a session of the CEA method was delivered to PPs while brief reminder with provision of pamphlet was used for the comparative group.

**Results:**

A total of 82 PPs were included in the analysis. The intervention group showed some positive trends in adherence especially in the use of sputum as first laboratory examination (RR = 1.24) and follow up (RR = 1.37), though not reaching statistical significance. After intervention PPs in CEA group maintained the adherence, but PPs in pamphlets group showed deterioration (score before to after: -12.5).

**Conclusion:**

Face to face education using CEA method seems to be as effective as brief reminder with provision of pamphlet in improving the adherence. CEA offers additional information that can be useful in designing intervention programs to improve the adherence to guideline.

## Background

Mycobacterium tuberculosis infects one-third of the world's population and imposes a global burden of an estimated 8 million new cases and 1.8 million deaths yearly [1]. More than 90% of global tuberculosis (TB) cases and deaths occur in the developing world, where 75% of cases are in the most economically productive age group (15-54 years). An adult with TB loses on average three to four months of work time [1,2].

Indonesia with a population of over 220 millions carries the heavy burden of TB. Indonesia still ranks third among the 22 high-burden countries [2]. In Bandung, one of the cities in West Java Province, Indonesia, case detection rate (CDR) did not reach the Indonesia target of 70% [3]. The Government of Indonesia considers TB control to be a high priority within the health-care system [3-5]. A strategy for incremental involvement of the private practitioners in DOTS (Directly Observed Treatment) implementation had been developed.

Private General Practitioners (PPs) are the first contact for TB patients. Their involvement is linked to the success of the TB control [6]. In Indonesia, it is generally believed that about a third of all TB cases might be partly or completely managed in the private sector [5]. Many studies indicated that PPs tended to deviate from recommended tuberculosis management guidelines [6]. Physicians' adherence with guidelines varies with different types of "patient" and with the length of clinical experience [6-10]. Adherence to program recommendations such as National Tuberculosis Programs (NTPs) is important for TB control.

Many strategies can be used to improve the adherence. Since clinical behavior is still a form of human behavior, psychological models of behavior change may be applied to modify practices of healthcare professionals [11-14].

Counseling, a face-to-face psychoeducation method can promote positive human interactions. The Catharsis Education and Action (CEA) method is a counseling technique that takes on many features of Carl Roger's person-centered psychotherapy. This method brings out the psychological concerns that result from wrong perception of reality and hinder appropriate behavior. These have been called emotionally critical misperceptions (ECMs). If addressed appropriately, barriers are lifted and educational inputs are better received. This method focuses not only on the problems but also on the opportunities for improvement and development [15-17]. As its name implies, the CEA method consists of three phases: catharsis, education and action. In the catharsis phase, the counselor spends time to clarify or define the problem. In the process, hidden emotions surface and ventilated so that they do not disturb the analytical functions of the mind [17-19]. In this phase, the PPs' problems in their management of tuberculosis patients are clarified and defined. Concerns about barriers and enablers to adherence to NTP (such as personal interest, patient choice and availability of diagnostic equipment and treatment) are addressed and explored. Through the utilization of active listening skills, genuineness, empathy and unconditional positive regard, one can accurately pinpoint and correct the most anxiety-provoking ECM. Once the ECM identified and corrected, it will now be easier to objectively analyze the problem. In this study, the discussion focused on identifying suspected TB patients, performing laboratory examination, treatment for TB patients, organizing follow ups, maintaining TB registries and DOTS implementation. It is in the education phase, that misperceptions are corrected using scientific evidence or the latest information available about the problem. It is presumed that appropriate behavior changes will be easier to accomplish after emotional burdens are released and new information and insight are provided. Implementation of the needed behavioral changes heralds the action phase [17].

## Methods

A randomized controlled trial was done to compare the effectiveness of the CEA method to the alternative method of brief reminders with provision of pamphlets on the management of tuberculosis.

The study was conducted in six primary health care centers in the Bandung District with the highest prevalence of TB. With the sampling frame composed of 288 PPs, the 86 PPs who met the inclusion criteria were randomized according to a computer generated randomization schedule. The inclusion criteria for the PPs were (1) registered in the District Health Office; (2) had patients with any of the following: features compatible with TB, sputum (+), chest X ray PA (+) and (3) willing to record and maintain TB registries. The allocation of PPs to either CEA or pamphlet group was done by concealed allocation wherein group assignments were coded and placed in sealed envelopes. Written informed consent was obtained from the participants of this study

### Intervention group

The intervention sessions were conducted in the PPs' practice. The first session was about twenty minutes. A protocol was developed to guide the investigator in conducting this method (Appendix 1). At this time, the PPs' problems in their management of tuberculosis patients were clarified and defined (**catharsis phase)**. The interactive case analysis and two-way communication for scientific evidence were employed in the **education phase**. After the education phase, the PPs committed to implement the NTP **(action phase)**. The second session was conducted for about ten minutes, three months after the first. At the sixth month, the outcomes were evaluated.

### Comparative group

In the comparative group, the PPs received a brief reminder with pamphlet on the management of tuberculosis and NTP protocols. The number of sessions and the evaluation of short-term effects were the same for the intervention group.

The primary outcome was adherence to tuberculosis guideline (NTP). Checklist for reviewing the patient's medical record was used by the investigator to assess adherence to NTP. This checklist covered the diagnosis of TB, treatment and follow up in accordance to NTP and the recording of treatment outcomes and all pertinent data such as all medications given, laboratory results, bacteriologic response, and adverse reactions (Appendix 2). This study assessed three medical charts in each practice to be chosen by the PP. Knowledge was assessed with a questionnaire adapted from a manual developed by the WHO in 2006 [20]. It covered knowledge on the diagnosis, treatment, follow up in accordance to NTP and treatment outcomes. The questionnaire was validated prior to the study.

The score for knowledge and adherence was calculated based on correct answer and performance for each item. The mean scores from both groups were used for the cut off point describing the PPs' knowledge and adherence. A score above the mean was regarded as good knowledge or adherence. Those who scored the cut-off point or above were considered as having good knowledge and the rest as poor knowledge. The Wilcoxon Signed Ranks test was used to assess the change of score before and after study in both groups. The level of significance was set at p < 0.05. Analysis was based on intention-to-treat.

## Results

**Figure 1:**
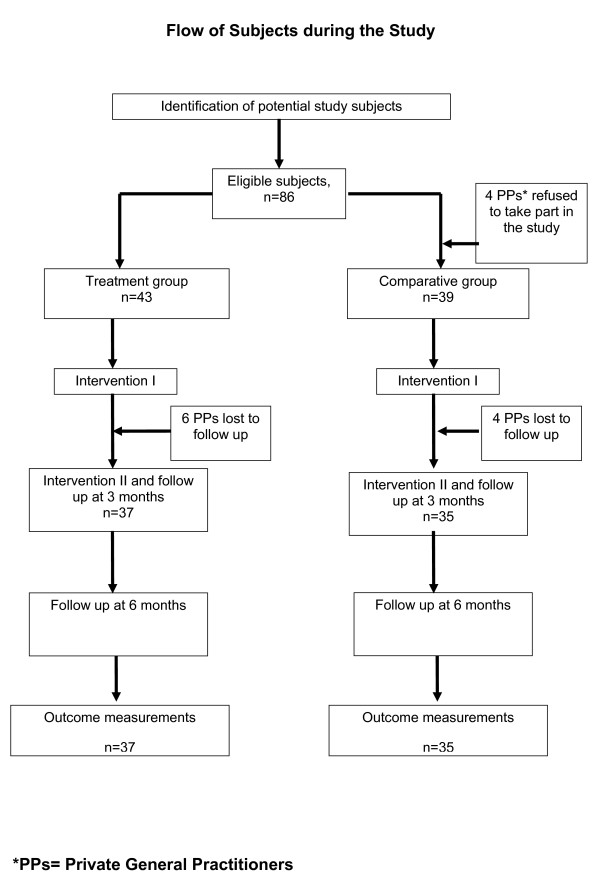
**Flow of the study**. This figure gives a brief description regarding the flow of study.

The data collection was done from September 1^st ^2007 until February 29, 2008. Of 86, four PPs refused to take part in the study (43 face to face education using CEA method group, 39 brief reminder using pamphlet group). All PPs who began an educational activity completed all learning activities, and tests. In the final evaluation six PPs lost to follow up in the intervention group and four PPs in the control group (Figure 1).

The PPs' characteristics are shown in Table 1. Thirty nine percent participants in CEA group and 38.5% in pamphlet group had attended training in tuberculosis and most of them attended once since they began practicing. Meanwhile, most PPs were not involved in the TB control program.

**Table 1:** Baseline Characteristics of Participants in the CEA and the Pamphlet Groups

Private general practitioner characteristics	CEA group (n = 43)	Pamphlet group (n = 39)	p-value
Mean age (SD)	32.42 (6.99)	42.15 (12.68)	< 0.001*

Sex, No (%)			0.075**
Male	18 (41.9)	24 (61.5)	
Female	25 (58.1)	15 (38.5)	

Number of training on tuberculosis attended			0.257**
• Less than 1	39 (90.7)	39 (100)	
• More than 1	4 (9.3)	-	

Provider, No (%)	n = 17	n = 15	0.491**
• Academic institution	5 (29.4)	3 (20)	
• District/Provincial Health office	11 (64.7)	12 (80)	
• Drug company	1 (5.9)		

Involvement in tuberculosis control program, No (%)			0.054**
Yes	10 (23.3)	3 (7.7)	
No	33 (76.7)	36 (92.3)	

Mean estimated number of TB cases treated per year since starting of practice (SD)	6.49 (9.4)	2.05 (2.75)	0.006*

Mean estimated number of TB patient completing the treatment per year (SD)	2.79 (7.76)	1.18 (1.6)	0.207*

Practice population	n = 31	n = 24	
• Social economic status, No (%)			0.295**
✓ Upper class	1 (3.2)	0	
✓ Middle class	8 (25.8)	3 (12.5)	
✓ Lower class	22 (71)	21 (87.5)	
• Educational level, No (%)			0.237**
✓ No school	3 (9.7)	0	
✓ Elementary school	13 (41.9)	9 (37.5)	
✓ High school	15 (48.4)	15 (62.5)	

* Independent t-test

** Chi-square test; Statistically significant at p ≤ 0.05

The study evaluated the knowledge and the adherence of PPs in both groups. The mean scores from the knowledge questionnaires were 65 for the CEA group and 76 for the pamphlet group. With these cut-off points, 51.2% of CEA group had good knowledge while 53.8% of the pamphlet group did. The corresponding cut-off points in adherence to guideline were 79 for CEA group and 70 for pamphlet group (Table 2). Half of PPs in both groups had good adherence to guideline.

**Table 2:** The knowledge and adherence of PPs in CEA group and pamphlet group

	Knowledge		Adherence	
	
	CEA group	Pamphlet group	CEA group	Pamphlet group
Mean score (SD)	64.95 (19.39)	76 (16.59)	79.17 (10.7)	69.64 (26.39)

Poor, no (%)	21 (48.84%)	18 (46.15%)	10 (47.62%)	9 (42.86%)

Good, no (%)	22 (51.16%)	21 (53.85%)	11 (52.38%)	12 (57.14%)

After the intervention, there was no improvement in the mean score of adherence to TB guideline in either group (Table 3). PPs in CEA group maintained the same mean score while PPs in pamphlets group showed deterioration though not of statistical significance.

**Table 3:** Change in median score of knowledge and adherence before and after intervention

	CEA group				Pamphlet group			
	
	Before n = 43	After n = 37	Change	p-value*	Before n = 39	After n = 35	Change	p-value*
Knowledge	71.43	92.86	21.43	**< 0.001**	78.57	92.86	14.29	**< 0.001**

Adherence	87.5	87.5	0	0.501	87.5	75	-12.5	0.096

* Wilcoxon Signed Ranks test

Statistically significant at p ≤ 0.05

Some concerns and problems on tuberculosis control were found during the CEA sessions. These included patient factors like socioeconomic background, stigma of TB in the community, and health seeking behavior; and from physician factors like experience, motivation, non-familiarity with guideline and lack of training.

## Discussion

The findings of this study contribute to the benefit of psychoeducational strategies in influencing physician behavior. At the individual level the CEA method and pamphlet produces better knowledge sustained in six months. Acquisition of knowledge was provided in this study using discussion and case analysis conducted twice within six months. Based on the Linear Model of Information Processing, for input to be transformed into long-term memory, the process of rehearsal such as repeating the case analysis is very much needed. The result of this process is knowledge retention.

This study showed that CEA method has the same effect as brief reminder with provision of pamphlet. The possible explanation for the minimal difference between the two groups might be the limited time spent to complete the CEA sessions for some PPs. For all PPs, concerns were elicited and addressed but time for education was occasionally shortened by the PPs themselves. Their busy schedule prompted the investigator to summarize the NTP education phase of the CEA session. In delivering education method to PPs, materials and methods needed to be adapted to their special needs and working conditions. At the baseline the PPs already had high adherence to NTP and in the six-month follow up they did not have enough more TB patients. This fact might account for no improvement in adherence to NTP. If the study was continued for a longer time, PPs would perhaps see more TB patients and showed more adherence to NTP. The other possible reasons were the characteristics of PPs such as training, experience in treating TB patients and involvement in the TB program.

During CEA sessions, PPs ventilated their reluctance to treat TB patient because of the high dropout rate. They also regarded themselves not having enough experience in treatment. Low motivation and lack of confidence were thus PPs' ECM in tuberculosis control. The CEA sessions brought to light these concerns and could help to improve PPs' adherence to NTP.

The deterioration in adherence to NTP in the pamphlet group might be due to the PPs' background. They were significantly older and saw less TB patients (Table 1). While longer years of clinical practice might be associated with more professional experience, routine work over many years might also tend to blunt the physician's readiness to accept new scientific evidence and consequently inhibit modification of practice.

### Limitation of Study

This CEA method was conducted in PPs practice setting where they should attend to patients as well. For some PPs, the CEA session was conducted in ten minutes only. It seems that for some PPs the intervention was less efficient and less effective because of the rather short time of intervention.

## Conclusions

Face to face education using Catharsis Education Action (CEA) method seems to be as effective as brief reminder with provision of pamphlet in improving the adherence to recommended national guideline on the management of tuberculosis patients (NTP). CEA offers additional information that could be useful in designing intervention programs to improve NTP adherence.

## Competing interests

The author declares that they have no competing interests.

## Authors' contributions

NA was the principal investigator of the study and involved in designing the study, supervising the data collection, reviewing/analyzing the data and writing the paper.
